# Intra-Arterial Chemotherapy with Doxorubicin and Cisplatin Is Effective for Advanced Hepatocellular Cell Carcinoma

**DOI:** 10.1155/2014/160138

**Published:** 2014-05-22

**Authors:** Ming-Chun Ma, Yen-Yang Chen, Shau-Hsuan Li, Yu-Fan Cheng, Chih-Chi Wang, Tai-Jan Chiu, Sung-Nan Pei, Chien-Ting Liu, Tai-Lin Huang, Chen-Hua Huang, Yu-Li Su, Yen-Hao Chen, Sheng-Nan Lu, Kun-Ming Rau

**Affiliations:** ^1^Division of Hematology-Oncology, Department of Internal Medicine, Kaohsiung Chang Gung Memorial Hospital, 123 Ta-Pei Road, Niaosong District, Kaohsiung 833, Taiwan; ^2^College of Medicine, Chang Gung University, Kaohsiung 333, Taiwan; ^3^Department of Diagnostic Radiology, Kaohsiung Chang Gung Memorial Hospital, 123 Ta-Pei Road, Niaosong District, Kaohsiung 833, Taiwan; ^4^Department of Surgery, Kaohsiung Chang Gung Memorial Hospital, 123 Ta-Pei Road, Niaosong District, Kaohsiung 833, Taiwan; ^5^Division of Hepato-Gastroenterology, Department of Internal Medicine, Kaohsiung Chang Gung Memorial Hospital, 123 Ta-Pei Road, Niaosong District, Kaohsiung 833, Taiwan

## Abstract

Advanced hepatocellular carcinoma (HCC) remains a fatal disease even in the era of targeted therapies. Intra-arterial chemotherapy (IACT) can provide therapeutic benefits for patients with locally advanced HCC who are not eligible for local therapies or are refractory to targeted therapies. The aim of this retrospective study was to analyze the effect of IACT with cisplatin and doxorubicin on advanced HCC. *Methods*. Patients with advanced HCC who were not eligible for local therapies or were refractory to sorafenib received doxorubicin (50 mg/m^2^) and cisplatin (50 mg/m^2^) infusions into the liver via the transhepatic artery. Between January 2005 and December 2011, a total of 50 patients with advanced HCC received this treatment regimen. The overall response rate (ORR) was 22% in all treated patients. In patients who received at least 2 cycles of IACT, the ORR was 36.7%, and the disease control rate was 70%. Survival rate differed significantly between patients who received only one cycle of IACT (group I) and those who received several cycles (group II). The median progression-free survival was 1.3 months and 5.8 months in groups I and II, respectively (*P* < 0.0001). The median overall survival was 8.3 months for all patients and was 3.1 months and 12.0 months in groups I and II, respectively (*P* < 0.0001). The most common toxicity was alopecia. Four patients developed grade 3 or 4 leukopenia. Worsening of liver function, nausea, and vomiting were uncommon side effects. This study demonstrated clinical efficacy and tolerable side effects of repeated IACT with doxorubicin and cisplatin in advanced HCC. Our regimen can be an alternative choice for patients with adequate liver function who do not want to receive continuous infusion of IACT.

## 1. Introduction

Hepatocellular carcinoma (HCC) is the sixth most common cancer and the third most common cause of death from cancer worldwide [1]. HCC is proportionately even more important in Asian countries than in the rest of the world. Indeed, approximately three-fourth of HCC cases occur in Asian countries due to the high prevalence of chronic hepatitis B virus (HBV) infection in the population [2]. Chronic HBV infection is a leading cause of HCC in most African and Asian countries with the exception of Japan [3]. The Asian Pacific Association for the Study of the Liver published consensus guidelines for the management of HCC in Asia [4].

In Taiwan, due to the high prevalence of HBV infection, the incidence of HCC was always the highest of all cancers, until recently it has been surpassed by colorectal cancer [5]. Nevertheless, HCC is still the most common cancer and the most common cause of cancer-related deaths in men in Taiwan. Late diagnosis combined with liver cirrhosis, high recurrence rates, high HBV DNA titers, and possibly genetic factors may all contribute to the poor prognosis of HCC in Taiwan [6].

The treatment of HCC is complicated by its highly variable biological behavior and the frequent coexistence of chronic liver disease, especially cirrhosis, in affected patients. Although surgery remains the most frequently employed treatment modality, curative resection is only possible in a minority of cases. Another curative treatment is ablation, which includes percutaneous ethanol injection, microwave coagulation, and radiofrequency ablation (RFA). These treatments have been widely performed on patients with small HCC, generally characterized by Child-Pugh A or B cirrhosis with fewer than three tumors, where each tumor is less than 3 cm in diameter [4]. For the large number of patients diagnosed beyond the criteria of curative resection or ablation therapies, palliative treatment may be the goal. At these stages, local therapies including RFA, alcohol injection, transarterial embolization (TAE), local radiotherapy, doxorubicin-eluting beads, and yttrium-90 microspheres are the available treatment options. For patients who are not eligible for local therapies, including patients with extrahepatic metastases and/or thrombosis in the portal vein or in its major branches, systemic therapies such as chemotherapy, targeted therapy, or intra-arterial chemotherapy (IACT) are the current treatment options [7].

The rationale for IACT is to maximize drug concentrations in the liver and in the target tumor, at the same time, to minimize systemic toxicities [8]. Fluorouracil (5-FU), doxorubicin, and cisplatin all showed activities against HCC [9] and can be given safely by intra-arterial infusion [10]. In our institution, HCC patients with portal vein thrombosis (PVT) and massive or diffuse infiltration of tumor as well as patients refractory to previous TAE or target therapy were recruited to be treated by IACT with doxorubicin and cisplatin together. In this retrospective study, we report the results of fifty patients who were treated by IACT with doxorubicin and cisplatin.

## 2. Materials and Methods

### 2.1. Ethics Statement

This retrospective study was approved by the Institutional Review Board (IRB) Committee at the Chang Gung Memorial Hospital, Kaohsiung, Taiwan. The written consent was specifically waived by the approving IRB.

### 2.2. Patient Eligibility Criteria

This study was a retrospective analysis of a clinical database of patients with advanced HCC who were treated by IACT at the Kaohsiung Chang Gung Memorial Hospital, Taiwan, between January 2005 and December 2011. The indications for IACT included thrombosis in the main portal vein or in the major branches of the portal vein, tumors refractory to previous TAE, and contraindications to TAE. Patients could receive either local treatments such as RFA or ethanol injection or systemic therapies before IACT. Inclusion criteria included an Eastern Cooperative Oncology Group (ECOG) performance status ≤2, with adequate organ and bone marrow function defined as absolute neutrophil count ≥1,000/mm^3^, platelets ≥50,000/mm^3^, aspartate aminotransferase (AST), and/or alanine aminotransferase (ALT) ≤5 times the upper limit of normal (ULN), bilirubin ≤2 mg/dL, and creatinine ≤1.5times ULN. Liver function had to be Child-Pugh class A or B. Patients with tumors with arterialvenous shunt were excluded from the analysis. Each patient received a computed tomography (CT) scan or magnetic resonance imaging (MRI) scan of the abdomen and pelvis. In addition, if lung metastasis was suspected, a chest CT scan was also performed.

### 2.3. Intra-Arterial Chemotherapy Procedures

Through a puncture site in the inguinal area, a catheter was inserted from the femoral artery to the celiac artery, and then to the proper hepatic artery. After selecting the major feeding artery of tumors, chemotherapeutic agents were injected sequentially into the tumors through the catheter, with the infusion rate controlled by an automatic infusion pump. After the injection of chemotherapeutic agents, the catheter was removed. The same procedure was repeated every time for IACT.

### 2.4. Chemotherapy Regimens

Both doxorubicin and cisplatin were given at a dose of 50 mg/m^2^. Doxorubicin was diluted in 100 mL normal saline, and the infusion time was 10 minutes. Cisplatin was diluted in 500 mL normal saline, and the infusion time was 3 hours. Premedications included dexamethasone, serotonin receptor antagonists, and adequate hydration. The procedure was repeated at 4–6 week intervals and was stopped at either disease progression, impaired liver function, severe side effects, or intolerance.

### 2.5. Evaluation of Response and Therapeutic Effects

Based on CT scans or MRI scans obtained before and after every two cycles of treatment, the response was evaluated according to RECIST criteria 1.1. Complete response (CR) was defined as the disappearance of all evidence of disease and the normalization of tumor markers for at least 4 weeks. Partial response (PR) was defined as a ≥30% reduction in unidimensional tumor measurements without the appearance of any new lesions or the progression of any existing intrahepatic lesion. Progressive disease (PD) was defined as any of the following: a 20% increase in the sum of the diameters of five measurable lesions, the appearance of any new lesions, or the reappearance of any lesion that had previously disappeared. Stable disease (SD) was defined as a tumor response that did not fulfill the criteria for CR, PR, or PD.

Progression-free survival (PFS) was defined as the time from the start of treatment until the date of clinical or radiological progression as determined by RECIST. Time to treatment failure (TTF) was defined as the time from the start of treatment to the date treatment discontinuation due to any cause, such as disease progression, side effects, or death. Overall survival (OS) was defined as the time from the start of second-line treatment until the date of death due to any cause.

### 2.6. Toxicity Assessment

Toxicity was assessed according to the National Cancer Institute Common Toxicity Criteria. Hepatic toxicity was defined as an increase in liver test results over the baseline values (3-4-fold for AST or ALT, and greater than 1.5-fold for bilirubin).

### 2.7. Statistical Analyses

The SPSS statistical package version 17 was used to process and analyze the data. Survival estimations were performed using the Kaplan-Meier method. The log-rank test was used for univariate analysis. Parameters with *P* values below 0.05 at the univariate level were entered into a Cox regression model in a stepwise forward fashion to analyze their relative prognostic importance. For all analyses, two-sided tests of significance were used and *P* values below 0.05 were considered significant.

## 3. Results

### 3.1. Patients Characteristics

From January 2005 to December 2011, a total of 50 patients with advanced HCC received IACT at the Kaohsiung Chang Gung Memorial Hospital. Forty-eight (96%) patients were male, and only 2 (4%) were female. The median age of patients was 52 years (range: 30–75 years). The etiology of underlying disease was HBV alone in 32 patients, HCV alone in 4 patients, both HBV and HCV in 11 patients, and alcoholism in 3 patients. Thirty-nine (68%) patients had liver cirrhosis at the time of diagnosis. Because of late diagnosis, only 8 (16%) patients had the opportunity to receive hepatectomy before. Most patients were BCLC stage C at the time of IACT. The major indications for IACT were thrombosis in the portal vein (PVT), followed by multifocal nodules that could not be covered by TAE, and TAE failure. Six (12%) patients had extrahepatic metastasis at the time of IACT, but as their dominant symptoms were due to the primary tumors, they were also deemed eligible for IACT. The median serum AFP value of patients was 307.9 ng/mL (range: 3.0–>87,500 ng/mL) (Table 1).

### 3.2. Treatment Results

**Figure 1:**
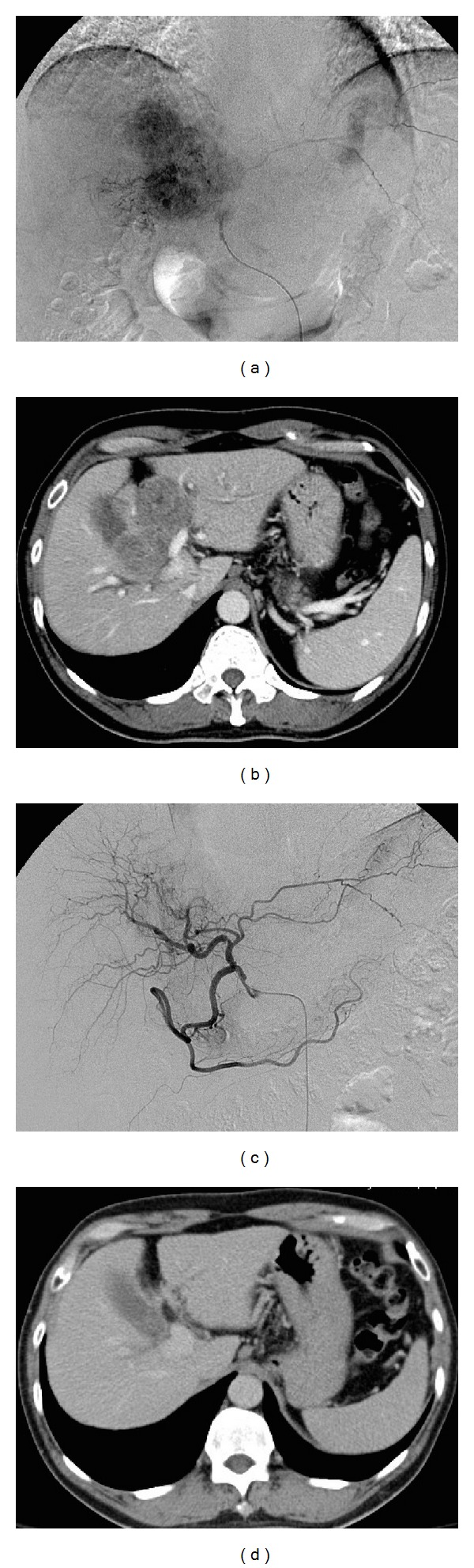
(a) Angiogram of the hepatic artery obtained before intra-arterial chemotherapy. (b) Computed tomography image of the liver obtained before intra-arterial chemotherapy. (c) Angiogram of the hepatic artery obtained after two cycles of intra-arterial chemotherapy. (d) Computed tomography image of the liver obtained after two cycles of intra-arterial chemotherapy.

Twenty patients received only one cycle of IACT (group I), ten of whom could not be evaluated for response. Thirty patients received at least two cycles of IACT (group II), including six patients who received more than four cycles. Although none of the patients achieved CR, the overall response rate (ORR) was 22% in all patients. In group II patients, the ORR was 36.7%, and the disease control rate was 70% (Table 2). There were no significant differences in the base line characteristics between patients in these two groups. The response to IACT could also be evaluated by changes in vascular density during angiography (Figure 1).

**Figure 2:**
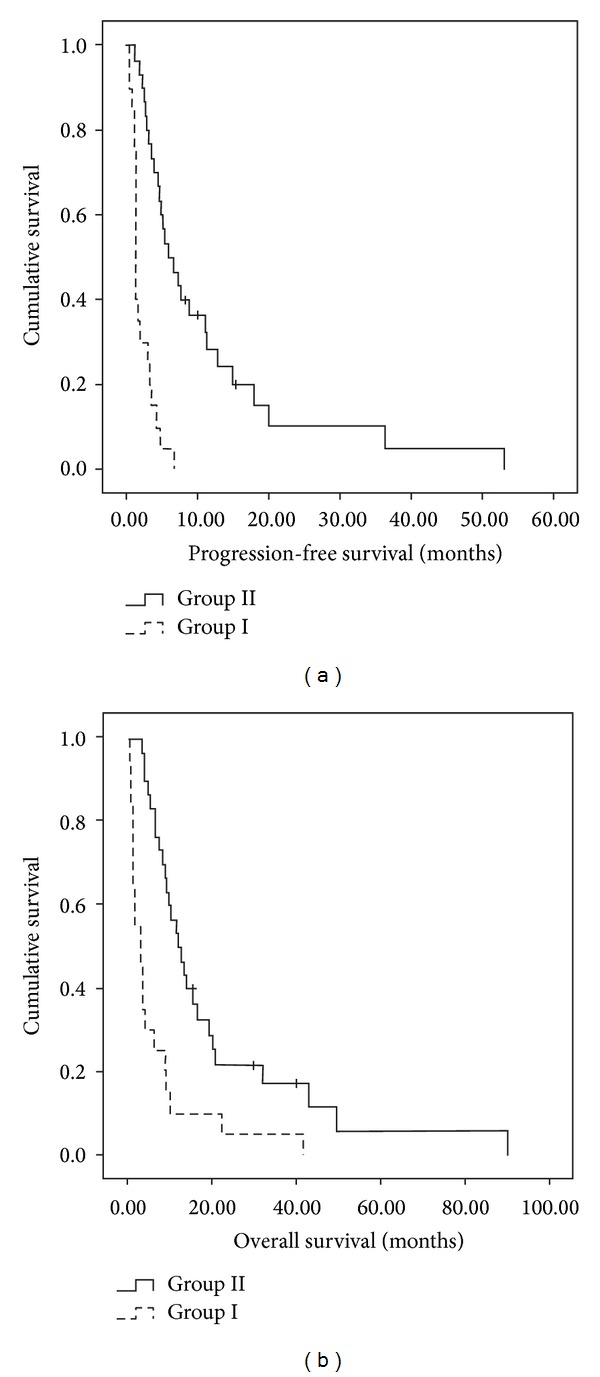
(a) Cumulative survival curve of progression-free survival. (b) Cumulative survival curve of overall survival.

Survival differed significantly between group I and group II patients. The median PFS was 3.6 months for all patients, 1.3 months for group I patients, and 5.8 months for group II patients (*P* value <0.0001). The median OS was 8.3 months for all patients, 3.1 months for group I patients, and 12.0 months for group II patients (*P* value <0.0001) (Table 3, Figure 2).

### 3.3. Toxicities

Four patients out of 50 developed grade 3 or 4 neutropenia during our study.

Even in patients who received more than 2 cycles of IACT, the incidence of grade 3 or 4 neutropenia was only 6.7%. Worsening of liver functions was uncommon, as was severe nausea or vomiting. The most common side effect encountered was alopecia (Table 4). In general, toxicities from IACT were deemed to be acceptable.

### 3.4. Reasons for Discontinuation of IACT

Tumor progression was the main cause of treatment discontinuation. Eleven patients stopped treatment due to adverse events, but only one patient was in the group who received at least two cycles of IACT. Six patients in our study group could receive local therapy after IACT, including two cases who received curative tumor resection (Table 5).

## 4. Discussion

For patients diagnosed with HCC, resectability is the most important factor determining cure. Resectability depends not only on tumor stage but also on the functional reserve of the liver before and after resection. Liver-confined HCC is often associated with large size, vascular invasion, or multifocality. Advanced presentation and underlying liver disease limit the application of curative options, but the unique blood supply of the liver provides a way for local therapies through the hepatic artery. Transarterial embolization, transarterial chemoembolization (TACE), doxorubicin-eluting beads, and radioembolization with yttrium-90 microspheres all can achieve long-term survival in some patients.

In Taiwan, viral hepatitis is the most common cause of liver cirrhosis and HCC. Delayed diagnosis is common, as reflected in our study by the high percentage (48%) of patients who did not have the opportunity to receive either liver resection or local therapies because of the presence of PVT. Other patients might have already received several treatments, and some of them had distant metastases at the time of IACT, which was the last chance treatment for them.

Although local therapies are effective against HCC restricted to the liver, there are still some contraindications for different therapies. These contraindications include major PVT, massive or diffuse infiltration of the tumor, poor liver function with Child-Pugh class C, and severe hepatic arteriovenous shunt. Before the era of targeted therapies, most patients with these contraindications were left with no further treatment options. Targeted therapies such as sorafenib are available at present, but their effect is still disappointing, especially in Asia-Pacific countries. In a randomized, phase III study of sorafenib, the median TTP was 2.8 months and the median OS was 6.5 months, but grade 3 or 4 side effects were common [22]. Another barrier for patients is the price of sorafenib, which is still too high to be affordable for most patients or organizations reimbursing the patients. Thus, for patients who cannot receive surgery, TAE, RFA, or targeted therapy, IACT is still one of the available choices.

The rationale for IACT is that increased local concentration of a drug is expected to result in increased therapeutic response, without high levels of systemic exposure to the given drug. Adriamycin, cisplatin, and floxuridine (5-fluoro-2′-deoxyuridine, FUDR) have been extensively used for IACT in HCC and other cancers [23–25]. In a phase III study in Japan, FUDR therapy resulted in a good response [12, 26], but disadvantages include prolonged infusion times and the need for a permanent arterial port catheter system in the femoral artery which may disturb patients in their daily activities. Some studies also combined the systemic administration of interferon-*α* to 5-fluorouracil treatment [19]. In summary, in several studies from Asia, chemotherapeutic agents such as 5-FU and cisplatin delivered into the hepatic artery via an implanted port system showed a favorable anticancer effect and improved response rates (Table 6). However, small sample size and lack of randomization generally make it difficult to recommend this kind of therapy for HCC with PVT [27]. In addition, protracted infusion of chemotherapeutic agents may have a negative impact on a patient's quality of life (QOL). Thus, it is desirable to tailor the treatment scheme to a shorter duration without compromising tumor response or increasing the incidence of adverse events. For patients with advanced HCC, cure is not the primary end point, survival benefit and QOL are. In our study, although the effect was only noninferior to other treatments, the time patients spent in hospital was relatively short. Shorter hospitalization can benefit patients by reducing the cost of treatment and by improving QOL.

In our treatment, we evaluated doxorubicin and cisplatin as therapeutic options for IACT. Both drugs can be given through the transarterial route with shorter infusion times compared to the protracted infusions of low-dose cisplatin and 5-FU which necessitate a relatively long-term treatment and hospitalization, as well as a permanent injection port implantation at the femoral site. In our current study, although the overall response rate (ORR) was only 22.0%, the median survival was 12.0 months and the median TTF was 7.0 months for patients who received at least 2 cycles of IACT; both were noninferior to other studies (Table 6). The infusion duration required for our protocol was only 3 hours for intra-arterial chemotherapy, and the catheter was removed immediately after IACT. The median cycle of IACT was 3; most patients tolerated repetitive puncture of femoral artery well. They could return to their daily activities soon after IACT. These aspects of our protocol make it cost-efficient and facilitate the task of caregivers. Compared to other studies, the major side effect was grade 3 or 4 neutropenia, which happened in 8% of all patients, but none of the patients died of sepsis, and most other side effects were manageable.

Multivariate analysis revealed that the most significant prognostic factor in our study was the number of IACT cycles that the patients received. The RR in patients who received at least 2 cycles of IACT was significantly better than in the intent-to-treat population (ORR 36.7% versus 22.0%). There were no major differences in the baseline clinical characteristics of group I and group II patients, including the tumor stage at IACT, Child-Pugh criteria, performance status, and hemogram. The median PFS in group I versus group II patients was 1.3 months versus 5.8 months (*P* < 0.0001), whereas the median OS was 3.1 months versus 12.0 months (*P* < 0.0001). The major cause for discontinuation was disease progression, which eventually resulted in the death of most of our patients. These results emphasized that patient selection should be more carefully evaluated before IACT with this regimen. Such patients who had diffusely infiltrated tumors, multifocal tumors, and borderline liver function might not receive IACT. Because most of our patients had extensive intrahepatic tumors, it was difficult to deliver the drugs to all the parts of the tumor, even if the tip of catheter was in the right place. Thus, the concentration of the drugs might have been diluted, mitigating the therapeutic effect in some patients. Another reason for discontinuation was rapid progression during IACT. Although severe leukopenia and impaired liver function were not so common after IACT, symptoms from tumor progression before they accepted the secondary cycle of IACT would worsen the general condition of our patients and make further treatment difficult.

IACT is not included in the guidelines of the European Society of Medical Oncology and only included in the National Comprehensive Cancer Network (NCCN) guidelines in the context of clinical trials [28]. However, the use of IACT is still a common practice in the treatment of advanced HCC in Asia.

## 5. Conclusion

The results of our study demonstrated clinical efficacy and tolerable side effects of repeated IACT with doxorubicin and cisplatin in advanced HCC. Since there is still no standard regimen for IACT, our regimen can be an alternative choice for patients with adequate liver function who do not want to receive continuous infusion of IACT.

## Figures and Tables

**Table 1:** Baseline clinical characteristics of patients.

	Number of patients	%
Sex		
M	48	96
F	2	4
Median age at diagnosis (year)	52 (range: 30–75)	
Hepatitis history		
HBV infection	32	64
HCV infection	4	8
HBV + HCV	11	22
Non-B, non-C	3	6
Liver cirrhosis		
Yes	39	78
No	11	22
Previous hepatectomy		
Yes	8	16
No	42	84
Previous local treatment*		
0	20	40
1	10	20
2	17	34
3	3	6
Previous systemic treatment**		
0	43	86
1	6	12
2	1	2
Child-Pugh class at IA chemotherapy		
A	42	84
B	6	12
C	2	4
BCLC stage at IA chemotherapy		
B	8	16
C	32	64
D	10	20
Indication for IA chemotherapy		
PVT	24	48
Multiple nodules	13	26
TAE failure	7	14
Extrahepatic metastasis	6	12
Pretreatment laboratory data, median (range)		
Bilirubin (mg/dL)		1.0 (0.4–2.0)
Albumin (g/dL)		3.4 (1.8–4.3)
Platelet count (3 × 10^3^/uL)		165 (54–589)
ALT (IU/L)		46 (16–184)
INR of prothrombin time		1.09 (0.93–1.33)
*α*-fetoprotein (ng/mL)		307.9 (3–>87,500)

ALT: alanine aminotransferase; BCLC: Barcelona Clinic Liver Cancer; HBV: hepatitis B virus; HCV: hepatitis C virus; IA: intraarterial; INR: international normalized ratio; PVT: portal vein thrombosis; TAE: transarterial embolization.

*Local treatments included transarterial embolization, alcohol injection, and radiofrequency ablation.

**Systemic treatments included chemotherapy, thalidomide, and targeted therapies.

**Table 2:** Treatment response.

Item	Number of patients	%
IA Cycle		
1	20	40
2	10	20
3	9	18
4	5	10
≥5	6	12
Response rate in all patients		
CR	0	0
PR	11	22
SD	11	22
PD	18	36
NA	10	20
Response rate in patients who received ≥2 cycles of chemotherapy		
CR	0	0
PR	11	36.7
SD	10	33.3
PD	9	30

CR: complete response; IA: intra-arterial; NA: not available; PD: progressive disease; PR: partial response; SD: stable disease.

**Table 3:** Survival data.

	All	Cycle = 1	Cycle ≥ 2	*P*
PFS				
Mean (months)	7.6	2.1	11.3	<0.0001
Median (months)	3.6	1.3	5.8	
OS from the start date of IACT				
Mean (months)	14.6	6.3	20.4	<0.0001
Median (months)	8.8	3.1	12.0	

IACT: intra-arterial chemotherapy; OS: overall survival; PFS: progression-free survival.

**Table 4:** Toxicity from treatment.

	Number	%
Grade 3 or 4 adverse events in all patients		
Neutropenia	4	8
Worsening of liver functions	2	4
Alopecia*	20	40
Nausea/vomiting	1	2
Grade 3 or 4 adverse events in patients who received ≥2 cycles of chemotherapy		
Neutropenia	2	6.7
Worsening of liver functions	1	3.3
Alopecia*	20	66.7
Nausea/vomiting	1	3.3

*Alopecia is grade 2.

**Table 5:** Reasons for discontinuation of intra-arterial chemotherapy.

Patients	Number	%
All patients	50	100
PD of primary tumor	22	44
PD at distant site	8	16
AE	11	22
Change to local therapy	6	12
Other	3	6
Patients who received ≥2 cycles		
PD of primary tumor	17	56.7
PD at distant meta	4	13.3
AE	1	3.3
Change to local therapy	6	20
Others	2	6.6

AE: adverse event; PD: progressive disease.

**Table 6:** Overview of studies using intra-arterial chemotherapy for HCC in Asia.

Investigator	Number of patients	Drug(s)	Efficacy	Major (grade 3 or 4) toxicity	Reference
Baek et al.	34	FUDR	RR: 41.2%; DCR 61.8%	Leucopenia (2.9%); thrombocytopenia (2.9%); gastric ulcer (11.8%); mucositis (8.8%)	[11]
Okusaka et al.	79 (TACE); 82 (IACT)	Zinostatin stimalamer	RR: 48.1% (TACE); RR: 34.2% (IACT)	Thrombocytopenia (3.7% in IACT group); AST (28%); ALT (20%)	[12]
Kim et al.	36	5-FU and cisplatin	RR: 16.7%	Thrombocytopenia (6.4%); hepatitis with ALT >2.5 times UNL (38.7%)	[13]
Mazzanti et al.	24	Folinic acid and 5-FU	RR: 54.2%	No	[14]
Chung et al.	23	IA cisplatin and SC INF-*α*	RR: 33%	NA	[15]
Park et al.	41	5-FU and cisplatin	RR: 22%; SD: 34.1%	Leukopenia (2.4%); thrombocytopenia (4.8%); bilirubin (12.2%)	[16]
Obi et al.	116	IA 5-FU and SC INF-*α*	RR: 51%	NA	[17]
Ando et al.	48	Cisplatin and 5-FU	RR: 43%	Hepatic dysfunction (8.3%)	[18]
Ota et al.	55	IA 5-FU and SC INF-*α*	RR: 43.6%	Leucopenia (5.5%); thrombocytopenia (9.1%)	[19]
Woo et al.	32 (low dose); 36 (high dose)	Cisplatin and 5-FU	Low dose RR: 0% High dose RR: 16.7%	Neutropenia (3.1% in low dose, 2.8% in high dose); hyperbilirubinemia (3.1% in low dose, 2.8% in high dose)	[20]
Tanioka et al.	38	Cisplatin and 5-FU	PR: 47%	NA	[21]
Rau et al.	50	Cisplatin and doxorubicin	RR: 22% (intent-to-treat) RR: 36.7% (>2 cycles) DCR: 70%	Neutropenia (8%)	This study
